# Fat chance for physical activity

**DOI:** 10.1186/1478-7954-11-9

**Published:** 2013-07-10

**Authors:** Bruce Neal

**Affiliations:** 1 The George Institute for Global Health, The University of Sydney, Royal Prince Alfred Hospital, Level 10, King George V Building, Missenden Rd, Sydney, NSW, 2050, Australia

**Keywords:** Obesity, Salt, Fat, Sugar, Tobacco, Government, Industry, Regulation

## Commentary

The enormous burden of ill health caused by overweight and obesity is well understood, particularly in developed countries like the United States, which have seen massive increases in prevalence over the last few decades [1]. Most recently the Global Burden of Diseases, Injuries, and Risk Factors Study 2010 reported that high body mass index (BMI) is now the third-leading cause of ill health in the United States, responsible for more than one-third of ischemic heart disease, half of osteoarthritis, and three-quarters of diabetes [2]. The consequences for the health, economics, and well-being of individuals and the community are enormous [3].

At its most basic level, obesity is easily understood as a problem of energy balance–if energy intake from food exceeds energy expenditure from physical activity, weight gain ensues. Likewise, it follows that if energy intake can be reduced or physical activity increased, weight loss should follow. Less clear has been the importance of each to the causation of the obesity epidemic and the potential for interventions targeting diet versus physical activity to reverse it. The paper by Dwyer-Lindgren et al. in *Population Health Metrics*[4] provides important new insight into where the United States should focus its efforts over the next few years.

The key observation in the report by Dwyer-Lindgren is the apparently paradoxical association between changes in physical activity and obesity–average levels of physical activity in the United States appear to have risen, but so too have obesity rates. The most likely explanation for this finding is that the rise in average physical activity levels has been outstripped by the excess of energy provided by the food supply. The message is plain – the primary driver of the obesity epidemic in the United States is now the food supply, and interventions targeting physical activity are not going to resolve it. So, while physical activity will benefit the health of the population even if it is not accompanied by weight loss [5], physical activity will not address most of the burden of ill health caused by obesity. That is going to require a new focus on the root cause of the problem –the American diet.

Before turning to consider what the solution might be, there’s one other consideration about causation that warrants mention–the obesity epidemic is not a problem that is caused by, or needs to be solved by, genetics. The obesity epidemic in the United States has arisen over just a few decades, a far shorter timeframe than even the most ardent geneticist could reasonably invoke for an evolutionary cause. While genetics may explain the reasons why some individuals suffer more or less severe consequences [6], it is transformation of the food environment that has driven the epidemic of obesity. The corollary to this is that it is environmental changes that offer some of the best, lowest cost, and most immediate opportunities to address the problem [7].

At the heart of the “energy in” side of the obesity problem is the food and beverage industry, which provides a continuous supply of processed and restaurant foods to most of the American population. Put simply, the enormous commercial success enjoyed by the food industry is now causing what promises to be one of the greatest public health disasters of our time. As fast as we rid the world of the microbial causes of pestilence and famine, they are replaced by new vectors of disease in the form of transnational food corporations that market salt, fat, sugar, and calories in unprecedented quantities [8].

The big question, of course, is how to turn the problem around. The push for higher levels of physical activity clearly should continue but will never be enough. Diet must be the new target and will require an approach that prioritizes renovation of the food environment above personal responsibility and individual behavior change. While individually targeted dietary interventions can reduce the weight of those that participate, delivery of intensive personalized interventions at scale will never be feasible because of the resources required. Furthermore, the magnitude of the weight reductions achieved with personal intervention have been disappointing, as have the rates at which weight is regained [9].

Changing the food environment will require a multifaceted approach that addresses both the average composition of foods available and how they are marketed [10]. Foods must be reformulated to have lower levels of salt, fat, and sugar and must be sold in smaller, less energy-dense servings. Marketing strategies must be controlled to better protect children, there must be standardized portion sizes, and there must be mandatory front-of-pack labeling that provides adults and children with easy-to-understand information about the likely effects of the product on their health. Policy makers must also commence work on pricing strategies that subsidize the cost of healthier foods, develop standards that define the need for warning labels on the least healthy products, and take actions to ameliorate the impact of upstream factors such as agricultural subsidies and trade agreements. This is a long and challenging list but it need not be daunting–policymakers should select the easiest one to start with and work up from there.

To control the obesity epidemic, government will have to take a much stronger leadership position. Industry can be a part of the solution to diet-related ill health but is too conflicted to contribute to policy development and should be engaged solely as the implementation partner [8]. The food industry will rail against the ‘’nanny state” and fight tooth and nail for its right to market a range of options to responsible individuals able to make choices for themselves–the American way. For context though, these arguments are no different to those used by the tobacco industry, which also markets habituating, unhealthy products in pursuit of profit. In the case of tobacco the American people have agreed that controls must be applied to limit the harms caused. Poor diet is now responsible for an even greater burden of disease than tobacco, and food companies must be controlled in the same way if the harms are to be reduced. As unpalatable as this may be, the food industry would do well to strengthen their public health conscience, given that consumers are always going to need their goods, something that cannot be said for tobacco and the industry behind it.

## Competing interest

BN is Chair of the Australian Division of World Action on Salt and Health. He has received consultancy fees from Pepsico (2011 and 2012) and holds a National Health and Medical Research Council of Australia Partnership Project grant (2010–2014) that includes contribution from the Australian Food and Grocery Council.
